# Enzymatic Oxidation of Ferulic Acid as a Way of Preparing New Derivatives

**DOI:** 10.3390/biotech11040055

**Published:** 2022-12-05

**Authors:** Abdulhadi Aljawish, Isabelle Chevalot, Cédric Paris, Lionel Muniglia

**Affiliations:** 1 Laboratory of Biomolecules Engineering (LIBio), Lorraine University, 2 avenue de la Forêt de Haye, TSA40602, F-54518 Vandœuvre-lès Nancy, France; 2 Laboratory of Reactions and Process Engineering (LRGP-UMR 7274), Lorraine University, 2 avenue de la Forêt de Haye, TSA40602, F-54518 Nancy, France

**Keywords:** enzymatic oxidation, LC-MS, RMN, antioxidant, anti-proliferative

## Abstract

The ferulic acid (FA)-oxidation by *Myceliophthora thermophila* laccase was performed in phosphate buffer at 30 °C and pH 7.5 as an eco-friendly procedure. LC-MS analysis showed that oxidation products were four dehydrodimers (P1, P2, P3, P5) at MM = 386 g/mol, two dehydrotetramers (P6, P7) at MM = 770 g/mol and one decarboxylated dehydrodimer (P4) at MM = 340 g/mol. Structural characterization showed that FA-dehydrodimers were symmetric for P1 and P5 while asymmetric for P2, P3 and P4. Physicochemical characterization showed that oxidation products presented a higher lipophilicity than that of FA. Moreover, symmetric dimers and tetra dimers had a higher melting point compared to FA and its asymmetric dimers. Antioxidant and anti-proliferative assessments indicated that enzymatic oligomerization increased antioxidant and anti-proliferative properties of oxidation products for P2, P3 and P6 compared to FA. Finally, this enzymatic process in water could produce new molecules, having good antiradical and anti-proliferative activities.

## 1. Introduction

The phenolic oxidation using enzymes to produce new compounds with interesting properties was vastly studied with a strong environmental concern [1]. In previous years, there have been several attempts to investigate the high potential of enzymatic oxidation in order to synthetize bioactive molecules from phenolic molecules, or to modify phenolic molecules with new structures such as monomers, dimers, trimers, tetramers, etchaving important properties such as antioxidant activity, anti-inflammatory, anti-cardiac effect, color, etc [1,2,3,4,5,6,7,8].

Polyphenol oxidases (PPO) are multi-copper, oxidative enzymes that can catalyze the oxidation reaction of phenolic derivatives to quinones producing different colors in vegetable injured tissues with the transformation of oxygen to water [9]. These enzymes include two enzymatic classes: tyrosinases and laccases. The tyrosinases (E.C. 1.14.18.1) contain a coupled binuclear copper active site. They can catalyze the hydroxylation of monophenols (cresolase activity) into *o*-diphenols and the two-electron oxidation of *o*-diphenols (catecholase activity) into free radicals (*o*-quinones) with the reduction of O_2_ into water [10].

Laccases (E.C. 1.10.3.2) that are blue multi-copper oxidases, contain a minimum of four copper atoms in their active site [9]. They catalyze the oxidation of phenolic compounds leading to the formation of corresponding free radicals, “quinones”, and reduce molecular oxygen to water. Moreover, they catalyze the oxidation reaction of monophenols into free radicals (semi-quinones) without the hydroxylation step in tyrosinase activity. Phenoxy radicals are electrophilic and reactive species that will undergo several chemical reactions with each other by covalent coupling to form new polymerized molecules such as dimers, trimers, oligomers and polymers by C-C, C-O and C-N bonds [11], or with active nucleophile groups such as the NH_2_ free amino groups of protein, polysaccharide or others [1,4,12,13,14].

Ferulic acid (FA), is a phenolic acid present in the plant as free form or esters [15], has many functional activities, such as antiradical, antimicrobial, anti-inflammatory, anti-thrombosis, and anti-cancer activities [16]. Several studies reported the characterization and the identification of oxidation products of FA in organic or aqueous medium using laccase enzyme [1,2,3,6,17]. In fact, FA-enzymatic oxidation forms semi-quinones as a reaction with each other to form oligomers (dimers, trimers, among others) by mainly covalent C-O or C-C bonds [8]. Previous work reported on the FA-enzymatic oxidation using laccase from *Trametes pubescens* in miscible solvents or hydro-organic medium to improve its antioxidant capacity [3]. Another study investigated the FA-enzymatic oxidation using laccase from *Myceliophthora thermophile* in biphasic system as an approach for the production of different colorants such as orange-yellow colorants [1,2,6]. Furthermore, the FA-enzymatic oxidation using laccase from *Rhus vernicifera* in aqueous medium (pH 7.4) was reported for the dyeing of silk, cotton, nylon, wool and viscose fabrics [8].

In the present study, the FA-enzymatic oxidation was performed at 30 °C and pH 7.5 in aqueous medium using *Myceliophthora thermophile* as a way of amplifying functional properties. In this study, the structural characterization of seven purified oxidation products was performed by HPLC, UN-Vis, LC-MS and NMR analysis and functional properties of each product were determined by lipophilicity (Log*P*), melting point, antiradical assay and anti-proliferative activity.

## 2. Materials and Methods

### 2.1. Chemicals and Enzyme

Ferulic acid (FA) and Trolox were obtained from Fluka (Paris, France); syringaldazine was purchased from Ega-chemie. Acetonitrile and methanol were obtained from Carlo Erba (Milwaukee, WI, USA). Trifluoroacetic acid (TFA) (98%) and 2,2′-azino-bis(3-ethlyl-benzthiazoline-6-sulfonic) acid (ABTS) were purchased from Sigma Aldrich (Paris, France).

The laccase enzyme (Suberase^®^, Perq, France) was purchased from Novozymes This enzyme is produced from *Myceliophthora thermophila* by the fermentation process of *Aspergillus oryzae*, which is genetically modified. This enzyme was obtained in the liquid form with a brown color and a density of almost 1.1 g/mL. According to method [2], this enzyme was purified to improve its activity.

### 2.2. Laccase Characterization

The enzyme protein was determined by spectrophotometric method using bicinchoninic acid (BCA) [18]. The protein standard used was Bovine Serum Albumin (BSA).

The laccase activity was measured using syringaldazine (200 µM) as a substrate in aqueous medium (phosphate buffer) at 50 mM and pH 7.5 using a spectrophotometer (Shimadzu UV-1605, Tokyo, Japan). This method is based on the increasing of absorbance at 525 nm caused by oxidized syringaldazine. The enzymatic unit was calculated as the substrate quantity (syringaldazine, µmole) oxidized per minute and per µg of protein [6].

### 2.3. FA-enzymatic Oxidation

FA oxidation was studied in a glass reactor in phosphate buffer (50 mM, pH 7.5) at 30 °C and 600 rpm. The reaction medium was composed of 10% FA solution at 50 mM and 90% of phosphate buffer. The reaction was started by adding 700 UI of laccase. A reaction without the laccase was done as a control. The FA-oxidation kinetics were monitored using High Performance Liquid Chromatography (HPLC). The enzymatic activity was stopped using a solvent (methanol + 0.03% of TFA (*v*/*v*).

### 2.4. Oxidation Kinetics by HPLC

The FA oxidation was monitored using HPLC (Shimadzu Class-VP HPLC, Tokyo, Japan). Separation of products was performed by LiChro-CART RP-18 column (Merck, 25 × 0.4 × 5 µm). The products were studied using a photodiode-array detector (PDA-M10A VP) at 20 °C without heating the column. Two solvents were used to realize the elution. Solvent A was composed from water and TFA (0.03; *v*/*v*), and solvent B was composed from 80% acetonitrile and 20% solvent A, with a flow rate of 0.7 mL/min. The gradient was used as follows: linear from 5% to 30% of solvent B for 14 min, from 30% to 55% of solvent B for 10 min and from 55% to 75% of solvent B for 10 min. Each analysis was made in triplicate.

### 2.5. Measurement of Reaction Mixture Color

The color of reaction medium was determined using a colorimeter (model 200) (Montreuil, France). The color parameters (L*, a*, b*) were determined where L* (lightness): black-white, a*: red-green and b*: yellow-blue [19]. The values of parameters were determined in triplicate. The color intensity (C) = (a*^2^ + b*^2^)^1/2^ was determined.

### 2.6. Characterization of FA Products

#### 2.6.1. Recovery of FA-Oxidation Products

The recovery of FA products was performed using a rotary evaporator under vacuum, and then a freeze-drying was performed lasting 24 h. Finally, the obtained products were stocked in a desiccator.

#### 2.6.2. Purification of FA-Products

The products were purified using a semi preparative HPLC. Separation of oxidation products was carried out using LiChroCART RP-18 column (Merck, 25 × 3 × 5). The products were detected at 280 nm using UV/Vis detector. The two solvents were used to separate the oxidation products: solvent A composed from water and TFA at 0.03 *v*/*v*), and solvent B composed from 80% acetonitrile and 20% solvent A using 5 mL/min as a flow rate. An amount of 1 mL of sample (10 mg/mL) was used as an injection volume. The used gradient was linear from 5% to 30% of solvent B for 15 min, from 30% to 50% of solvent B for 15 min and from 50% to 75% of solvent B for 15 min.

#### 2.6.3. Liquid Chromatography-Mass Spectrometry (LC-MS)

LC-MS system (LTQ-MS) was used to assess the mass spectra at atmospheric pressure ionization interface operating in Atmospheric Pressure Chemical Ionization (APCI) positive mode using LiChroCART RP-18 column (Merck, 25 × 0.4 × 5 µm). The spray voltage was used at 6.0 kV. The temperatures of the APCI vaporizer and of the heated capillary were used at 400 °C and 225 °C, respectively. The flow rates of auxiliary gas, sweep gas and sheath gas were determined at to 5, 5 and 48 (units/min), respectively. A voltage of 50 V was used in tube lens, 1 kV was used as capillary voltage and the values of front lens and split lens were −6.75 V and 70 V, respectively. To optimize all parameters, the FA solution at 0.1 mg/mL was infused using two solvents (solvent A was composed from water and TFA (0.03%)/solvent B was composed from 80% acetonitrile and 20% solvent) with a flow rate of 5 µL × min^−1^. The full scan was measured from 50 *m*/*z* to 1000 *m*/*z*.

#### 2.6.4. Nuclear Magnetic Resonance Spectroscopy (NMR) Analysis

^1^H and ^13^C NMR spectroscopic analysis was used to study the chemical structure of the oxidation products in dimethyl sulfoxide (DMSO) using a Bruker 300 spectrometer (Graetz, Germany) (300.13 MHz, 25 °C). The following notations were used: s: singlet, t: triplet, m: multiplet, br: broad, bold data: data used for the structural elucidation of oxidation products.

### 2.7. Physical Properties of Oxidation Products

#### 2.7.1. Lipophilicity (log*P*)

Tested molecules were prepared in 1-octanol at 0.3 mM, and then heated to 60 °C for 1 h to improve the solubility. The UV spectrum and the maximal absorbance (A0) were determined. Each organic solution was mixed with phosphate buffer (0.1 M, pH 7.4) at a ratio of (1:1) for 1 min. The mixture was left for 30 min to separate well (organic phase and aqueous phase). The absorbance of the organic phase was obtained (Ax). The partition coefficient (log*P*) was obtained according to following Equation (1):

*P* = Ax/(A0 − Ax) (1)

Each test was carried out in triplicate and the results were shown as mean values with standard deviation.

#### 2.7.2. Measurement of Melting Point

The melting point of FA and its products was determined using a melting device (BUCHI, Sankt Gallen, Switzerland). The measurement conditions were 100–240 V, frequency 50/70 Hz and power 150 W using a 5 µL capillary tube. Each test was performed in triplicate and the results were shown as mean values with standard deviation.

### 2.8. Antiradical Properties Using ABTS

The ABTS radical scavenging activities of tested molecules were performed according to a previous study [2]. The ABTS**^+^** solution was prepared by mixing 2.45 mM potassium persulfate and 7 mM ABTS, and it was then stored in the dark at room temperature for 12–16 h before use. In this work, the ABTS**^+^** solution was diluted with ethanol at room temperature up to an absorbance of 0.700 ± 0.025 at 734 nm. Each sample was prepared as follows: 10 µL of tested molecule at different concentrations (from 0.5 µM to 50 µM) were added to 1 mL of diluted ABTS**^+^** solution and this solution was then homogenized and stored in the dark at room temperature for 15 min. The ABTS**^+^** absorbance at 734 nm was determined using an ethanol as a blank. ABTS**^+^** radical scavenging activity was calculated by following Equation (2):

ABTS**^+^** radical scavenging activity (%) = (1 − Abs_sample_/Abs_control_) × 100 (2)

ABTS**^+^** radical scavenging activity was calculated as the half-maximal inhibition concentrations (IC_50_) and Trolox Equivalent Antioxidant Capacity (TEAC) values. Each test was done in triplicate and the results were shown as mean values with standard deviation.

### 2.9. Anti-Proliferative Activity

#### 2.9.1. Cells and Cell Culture

Colonic endothelial tumor (Caco2) cells were colonic endothelial tumor cells obtained from the laboratory URAFPA (Nancy, France). They were tested from passages 50 to passages 60 and cultivated in Dulbecco’s modified eagle medium (DMEM) with 4.5 g/L of glucose (Sigma, Neustadt, Germany) and 10% fetal calf serum obtained from EuroBio, (Les Ulis, France). L-glutamine and nonessential amino acids obtained from GIBCO, (Waltham, MA, USA) were used at 2 mM and 1%, respectively. After 2 days, the cells were rinsed with Dulbecco’s phosphate-buffered saline (D-PBS) (Sigma, Germany) and then trypsinized with trypsin enzyme (0.25%). For maintenance, the cells were seeded at 2 × 10^4^ cells/cm^2^ in flasks.

#### 2.9.2. Determination of Cell Viability

The FA-cytotoxic activity and its products was studied using Caco2 cells. A total of 5 × 10^4^ cells/well prepared in 195 µL of culture medium were seeded. After 24 h, 5 µL of the tested molecules at different concentrations (from 0.5 µM to 10 µM) were added to each well and then the microplate was incubated for 48 h at 37 °C, under 5% CO_2_ atmosphere. In each microplate, two columns were used as controls without tested molecules.

After 48 h of incubation, the Caco2 cells were rinsed with D-PBS and then, 200 µL of neutral red solution (50 µg/mL) was added at each well. After 3 h of incubation at 37 °C, the attached cells were rinsed with D-PBS and then solubilized in 200 µL of ethanol/water/acetic acid solution (50%, 49%, 1%, *v*/*v*/*v*). Finally, the microplate was shaken for 10 min at room temperature. The absorbance at 540 nm was done using a microplate reader (multiscan spectrometer, Thermo Scientific, Villebon sur Yvette, France). The relative cell viability was determined as the following Equation (3):

Relative cell viability (%) = ((1 − (Abs_treated cell_/Abs_control_)) × 100 (3)

Each test was performed in six copies, and in triplicate. IC_50_ mean values with standard deviation were calculated. IC_50_ was defined as the concentration of a molecule causing 50% cell mortality.

### 2.10. Statistical Analysis

All tests were determined in triplicate. The data were calculated as mean values with standard deviation (SD) and analyzed using SPSS (version 11.5.). ANOVA procedure was used for one-way analysis of variance. Duncan’s Multiple Range tests were used to calculate significant differences. Differences at *p* < 0.05 were considered significant.

## 3. Results and Discussion

### 3.1. Characterisation of Laccase

The bicinchoninic acid (BCA) method indicated that laccase solution contains 9 mg/mL of protein. The laccase enzyme showed its best activity at 30 °C and pH 7.5, using syringaldazine as the specific substrate. Under these parameters, the specific activity of laccase was determined to be 0.3 µmol/min/µg of enzymatic protein (UI).

### 3.2. Oxidation Kinetics

FA-oxidation kinetics were performed. Results indicated that the oxidation of FA was completed after 6 h of reaction time. Furthermore, this oxidation was not done without laccase. After 6 h, added novel FA was not oxidized, indicating that laccase was not inhibited due to the blockage of the active site of the enzyme by oxidation products [20]. In fact, the reaction of free radicals with the histidine groups of the enzyme active site is the main reason for enzyme inactivation [20].

### 3.3. Color Measurement

During the enzyme oxidation of FA in phosphate buffer, the color of reaction mixture changed. By qualitative analysis, the reaction medium color changed from colorless to orange-yellow at 3 h and then the reaction mixture color became brown at the complete consumption (at 6 h) of FA according to previous results [2,6]. By quantitative analysis, L* values of FA-oxidation medium decreased from 25.2 at 0 min, to 18.1 at 6 h. Moreover, at the start of the reaction, the values of red (a*) and of yellow (b*) simultaneously increased from *a* = −0.25 and *b*= −1.5 at 0 min until *a* = 2.1 and *b* = 3.3 at 3h. Then, the values of a* and b* decreased to 1.2 and 1.9, respectively at 6 h. These findings indicated a decrease in red and yellow color mainly due to the increase in polymerization degree of the oxidation products. At the final values of a* and b*, the reaction mixture of FA presented a high color saturation (C*) (27.4 ± 0.60) corresponding to brown color. In fact, in aqueous medium, the oxidation and polymerization rate of FA is very fast due to the high activity of both enzymes and free radicals. Therefore, the enzymatic oxidation in hydro-organic medium was reported as a way of reducing of polymerization degree by transferring the oxidation products into the organic solvent [21].

### 3.4. Characterization of Laccase-Catalyzed Oxidation Products

#### 3.4.1. HPLC Analysis

**Figure 1:**
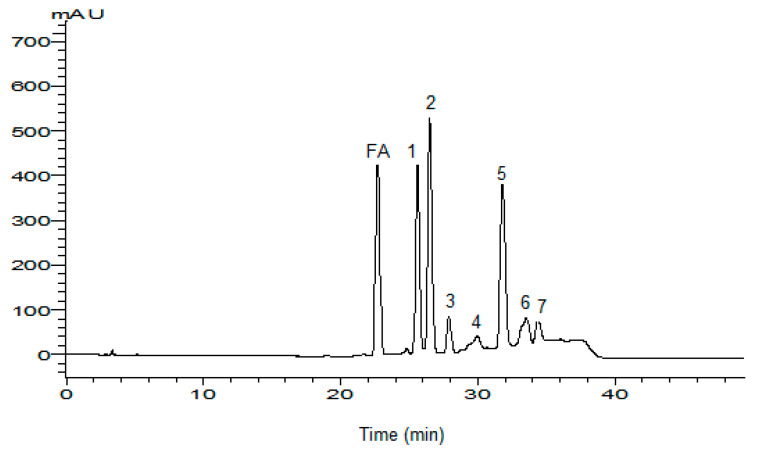

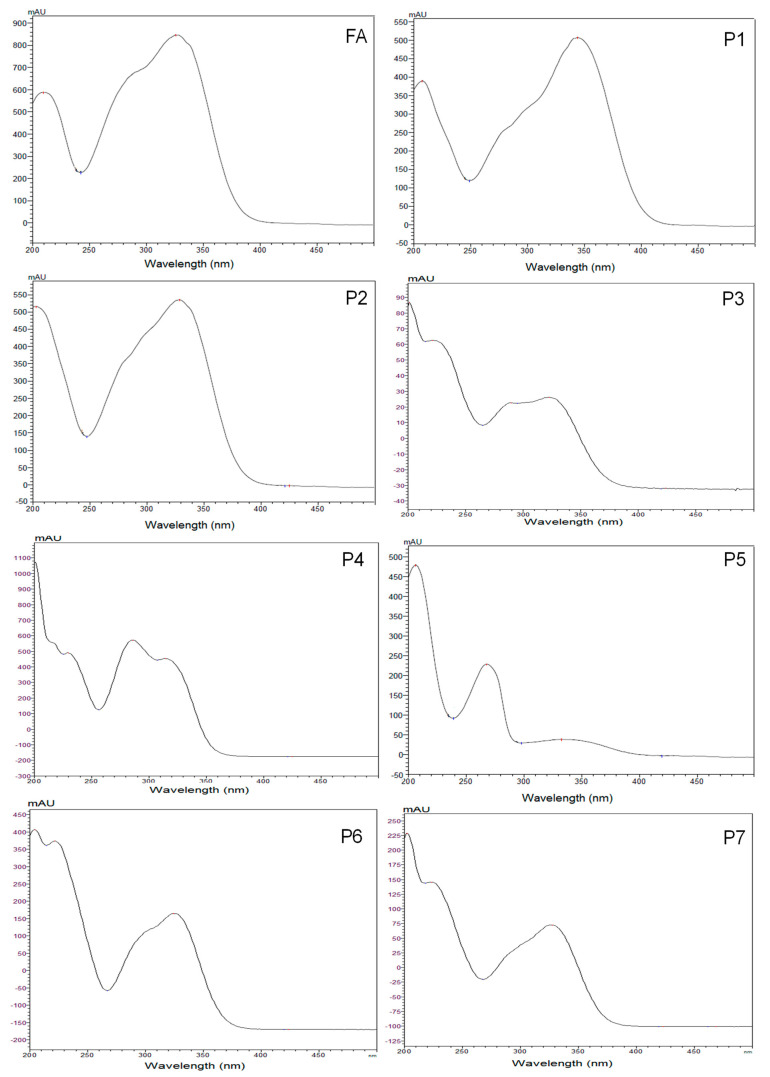
Reaction mixture chromatogram of FA after 3 h of reaction time and UV-spectra collected by HPLC-DAD of FA and its purified oxidation products. Column: RP-18, eluent: 80% acetonitrile and 20% water/TFA (20:0.03; *v*/*v*), flow rate: 0.7 mL min^−1^. λ = 322 nm.

HPLC analysis of FA products after 3 h showed several peaks which correspond to several products as presented in Figure 1.

The identification of FA products was done on seven main products. For the retention time (Rt), the major products were named P1, P2, P3, P4, P5, P6 and P7 corresponding to Rt: 25.7, 26.5, 28.1, 29.9, 31.9, 33.4 and 34.5 min, respectively, while FA is at 22.4 min. All oxidation products were released after FA in the reversed phase column (C18), indicating that the hydrophobic properties of oxidation products are higher than that of FA.

The UV spectra of FA and its oxidation products obtained by HPLC were shown in Figure 1. It was found that UV spectrum of FA presents a maximal absorbance at 322 nm due to the ethylene double linkage of the propionic chain and at 280 nm due to aromatic cycle. In a previous study, it was shown that the oxidation products showed a high absorbance at 322 nm and a low absorbance at 280 nm, [2] due to the violet-shift of the absorption range. The intensity decrease of the distinguishable long-wave bands were ascribed to the dissociation of carboxylic groups, which belong to a conjugated system [17]. By the comparison between the absorbance at 322 and 280 nm, the products (1, 2, 6 and 7) showed a high absorbance at 322 nm, while the product (P4) showed a high absorbance at 280 nm. The absorbance at 322 and 280 nm was almost the same for P3, whereas P5 showed absorbance at only 280 nm, indicating the absence of the ethylene double linkage of the propionic chain.

#### 3.4.2. Purification of FA-Products

For 100 mg of ferulic acid, the quantity of products obtained was 26 mg of P1 (26%), 31.5 mg of P2 (31.5%), 7.3 mg of P3 (7.3%), 6.2 mg of P4 (6.2%), 17.5 mg of P5 (17.5%), 3.6 mg of P6 (3.6%), 2.7 mg of P7 (2.7%) and 5.2 mg of unknown products.

#### 3.4.3. LC-MS Analysis

**Figure 2:**
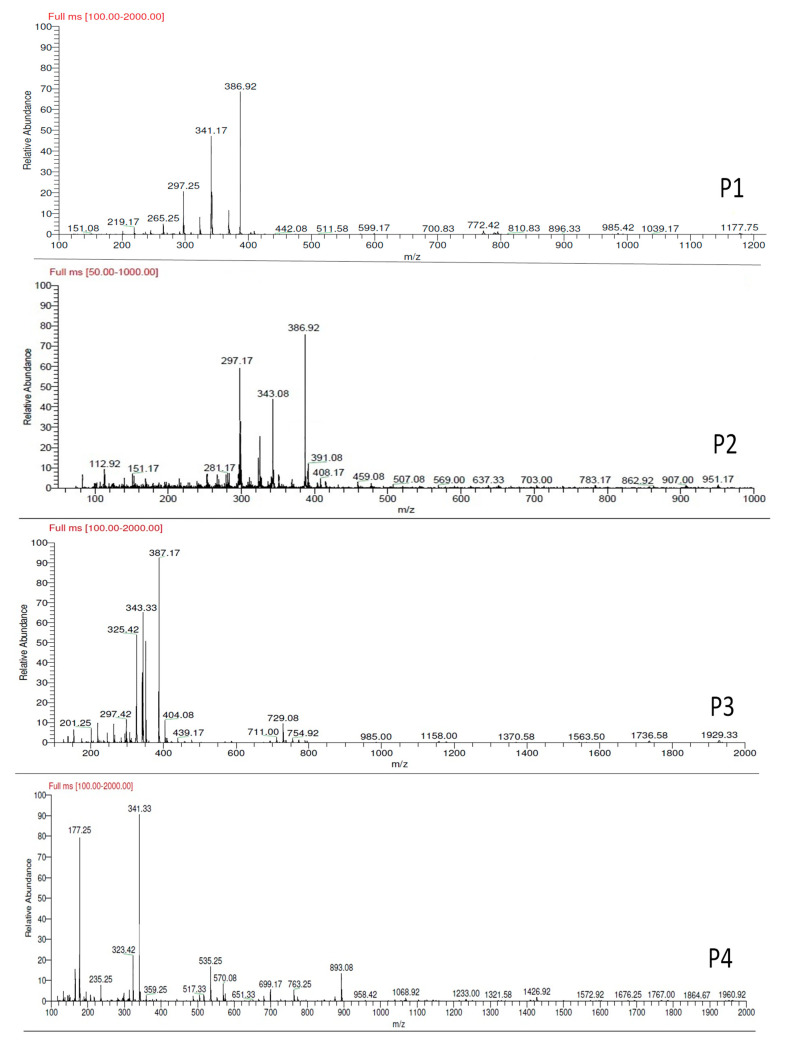

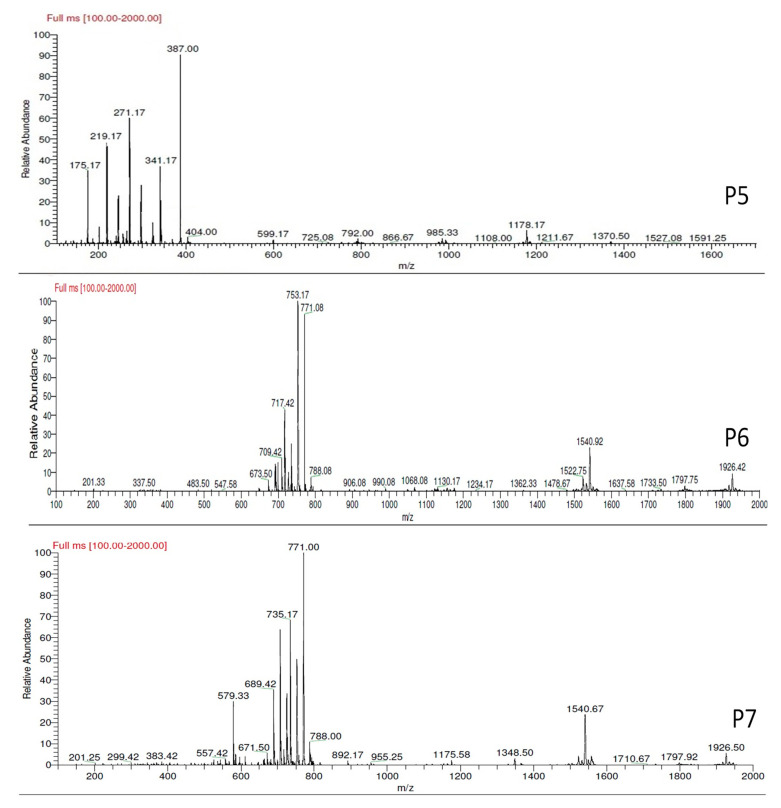
LC-MS spectrum (Full scan) of FA and its products in positive ion mode.

The LC–PDA-MS analyses in APCI positive ion mode of FA-laccase catalyzed oxidation products were performed (Figure 2). Results revealed that the products observed in UV led to *m*/*z* = 387 for P1, P2 and P3, corresponding to FA-dehydrodimers [M-H]^+^. The ion having *m*/*z* = 404 for P5 corresponds to the FA-dimer having *m*/*z* = 387, which is related with the water molecule (H_2_O) as explained by another study [4,22]. These authors showed the possibility of nucleophilic attachment of a water molecule on the FA-tetrahydrofuran depending on the reaction pH. Another study showed the possibility of the loss or addition of a water molecule on a tetrahydrofuran dimer of caffeic acid during LC-MS analysis [23]. Moreover, the ion having *m*/*z* = 341 for P4 corresponds to decarboxylation of the FA-dimer (*m*/*z* = 387) leading to [M-CO_2_-H_2_]^+^. Furthermore, the MS peaks on a full scan of P6 and P7 were found to be at *m*/*z* = 771, which corresponds to dehydrotetramers consisting of two FA-dimers (*m*/*z* = 387), leading to [2M+H-H_2_]^+^ as explained by another study [4]. The presence of other ions on full scan MS was related to natural fragmentation due to the high temperatures used in the APCI vaporizer and in the heated capillary (400 °C and 225 °C, respectively).

Several studies reported the identification of FA products using the laccase enzyme in aqueous and organic media. Previous work reported the formation of FA-dimers at MM = 386 (g/mol) using laccase from *Pyricularia orysae* at 37 °C and pH 6 in acetate buffer with 45% ethanol [17]. Another study confirmed the formation of FA-dimers at MM = 386 and 340 g/mol using laccase from *Myceliophthora thermophile* at 30 °C in phosphate buffer (pH 7.5) with 80% ethyl acetate [6]. Moreover, two FA-dimers at MM = 386 g/mol, one FA-trimer at MM = 580 g/mol and one FA-dehydrotetramer at MM = 770 g/mol were formed using laccase from *Trametes pubescens* (pH 5 at 28 °C) in acetate buffer with 80% ethyl acetate [3]. Sun and his colleagues reported the identification of three FA-dimers in aqueous medium (phosphate buffer), pH 7.4 at 40 °C using laccase from *Rhus vernicifera* [8]. Another study on the coupling of chitosan glucosamine with phenol using laccase from *Myceliophthora thermophila* confirmed that the FA-dimer at 341 g/mol was linked to glucosamine. Recently, the coupling of ferulic acid with two inflexible globular proteins (lysozyme and ovalbumin) by *Trametes versicolor* laccase was reported. In this study, ferulic acid was coupled with protein as two different types: dehydro-diferulic acid at a molar mass of 386 g/mol and dehydro-decarboxylated-diferulic acid at a molar mass of 340 g/mol [4].

LC-MS results showed that FA-enzymatic oxidation led to a formation of dehydrodimers at MM 386 g/mol, decarboxylated dimers at MM 340 g/mol and dehydrotetramers at MM 770 g/mol formed by coupling two dehydrodimers (MM 386 g/mol).

#### 3.4.4. NMR Analysis

**Figure 3:**
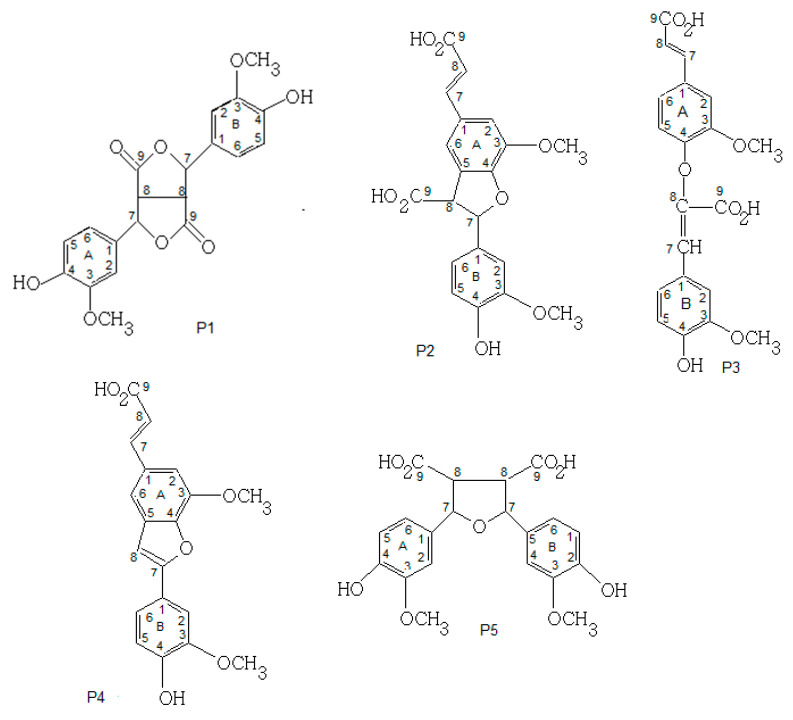
Dehydrodimers (P1, P2, P3, P4, P5) formed during FA oxidation in phosphate buffer at pH 7.5 and 30 °C.

The RMN analyses of FA products were performed for five FA-dehydrodimers (P1, P2, P3, P4 and P5) as shown in Figure 3. Two FA-dehydrotetramers were not analyzed due to their insufficient quantity.

NMR results showed that P1 and P5 at MM = 386 g/mol are symmetric dimers of two FA-monomers covalently bound through a C_8_-C_8_ linkage (Table 1). The symmetry of the molecules P1 and P5 facilitated assignment due to the clear signals and separation from each other. The signals in ^1^H- and ^13^C-NMR showed only seven kinds of hydrogens and ten kinds of carbons, confirming the symmetric structure. Consequently, P1 was found to be consisting of two equivalent tri-substituted aromatic rings, two equivalent saturated parts and two methoxy groups; in contrast, P5 had two equivalent tri-substituted aromatic rings, two equivalent saturated parts, two methoxy groups and two carboxylic groups. Several studies identified the P1 dimer of FA under the name 8-8′-di-lactone during the oxidation of FA using peroxidase and H_2_O_2_ in aqueous medium [24]. Moreover, this FA-dimer was identified during FA oxidation in aqueous and organic media [3,8,17]. The P5 dimer has been isolated from plant cell walls and identified under the name 8-8′-tetrahydrofuran [22]. As two (P1, P5) dimers of the five dehydrodimers identified result from C_8_-C_8_ radical coupling, this mechanism of radical coupling is favored by FA chemistry according to a previous study [24].

Furthermore, P2 and P3 at MM = 386 g/mol were asymmetric dimers of two FA-monomers covalently bound through a C_8_-C_5_ and a C_8_-O-C_4_ linkages, respectively. Moreover, P4 at MM = 340 g/mol was an asymmetric dimer obtained by decarboxylation of P2 (M-COOH, -H) covalently bound through a C_8_-C_5_ linkage. As expected, the spectra for P2, P3 and P4 were very hard to identify due to a lack of symmetry in the compound (Table 2). Consequently, the P2 dimer was found to be consisting of one tri-substituted aromatic fragment B, one tetra-substituted aromatic fragment A, one substituted double bond, a saturated fragment, two carboxylic groups and two methoxy groups. The P3 dimer contained two non-equivalent tri-substituted aromatic rings, two-substituted double bonds, two carboxylic groups and two methoxy groups. The P4 dimer had the same structure of P2 but without the carboxylic group. Several works identified the P2 dimer of FA in the cell walls of various plants [25]. In grass cell walls, one dehydrodimer was identified under the name 8-5′-benzofuran DiF. This FA-dimer was the major dehydrodimer produced during FA-peroxidase-catalyzed oxidation [24]. As this FA-dimer was produced by FA oxidation catalyzed by laccase or peroxidase, the FA structure enhances the formation of this dehydrodimer (C8-C5), irrespective of employed enzyme and irrespective of whether the reaction was performed in vitro or in vivo [24]. The P4 structure has been identified as the decarboxylated form of the P2 dimer (8-5′-benzofuran DiF) during lignin peroxidase-catalyzed oxidation of FA [24]. Indeed, the decarboxylation of FA-dimers by lignin peroxidase indicated that the identified dehydrodimers are intermediates in the polymerization reaction of FA. The P3 structure has been identified as a FA dehydrodimer during FA oxidation by laccase in phosphate buffer medium [8,17].

Based on the LC–MS and NMR results, Figure 3 proposes the final chemical structures of the FA-dehydrodimers using C-C and C-O-C radical coupling.

### 3.5. Physic Properties of Oxidation Products

#### 3.5.1. Lipophilicity (Log*P*)

Log*P* was performed for FA-laccase-catalyzed oxidation products in an n-octanol solvent in the presence of water (Table 3) (*p* ˃ 0.05). It was noted that a higher log*P* value represents a higher hydrophobicity [26]. The results revealed that the hydrophobicities of FA products are higher than that of FA, especially for the dehydrotetramer products, confirming the HPLC results. Previous work reported that the Log*P* value of FA was found to be of 1.5, confirming our results [27].

#### 3.5.2. Melting Point

Results revealed that melting point of FA was higher than that of the P2, P3 and P4 products, while it was lower than that of the P1, P5, P6 and P7 products (Table 3) (*p* ˃0.05). These results are due to the intermolecular forces between atoms of symmetric (P1, P5 and P6, P7) products are stronger than those of FA and asymmetric (P2, P3, P4) products. In fact, the melting point of FA was found to be between 170–174 °C, confirming our results. In the literature, it was reported that a major factor which determines the melting points is rotational symmetry. Moreover, symmetric molecules have high melting points due to the high force of attraction between atoms compared to asymmetric molecules [28].

### 3.6. Antioxidant Properties of FA Products

Results revealed that the FA molecule presented higher antiradical activity than Trolox, according to previous data obtained by other studies [29]. Additionally, the oxidation products P2, P3, P6 and P7 presented higher antiradical activity compared to the Trolox molecule, while products P1, P4 and P5 had lower antiradical activity in comparison with Trolox (TEAC value higher than 1). Furthermore, the enzymatic oxidation of FA improved the antiradical activity of products P2, P3 and P6 compared with FA. Nevertheless, products P1, P4 and P5 presented lower antiradical activity compared to FA. One product (namely P7) exhibited a similar antiradical activity as FA.

In fact, the antioxidant activity of phenolic acids as FA toward ABTS radical depends on the chemical structure of molecules such as the OH bond [30]. This activity is due to *ortho* substitution with the electron donor methoxy group, which increases the stability of the phenoxy radical [31]. The increase in antioxidant capacity of FA-dimers (P2, P3, P6) is due to an increase in functional groups, which give an electron after the dimerization process [32], and to the carboxylic group with an unsaturated C-C double bond which can assure additional attack sites for free radicals [33]. The reduction in antioxidant capacity of FA-dimers (P1, P4, P5) can be mainly due to the loss of the unsaturated C-C bonds and carboxyl groups during the dimerization process.

Consequently, the FA-enzymatic oligomerization allowed for improved ABTS radical scavenging activity for FA-asymmetric dimmers (P2 and P3), and reduced it for FA-symmetric dimmers (P1 and P5) in comparison with the FA molecule. These data are in accordance with the results obtained for FA-enzymatic dimerization in acid and solvent medium [3]. Another work confirmed that enzymatic oxidation of phenols such as ferulic acid using tyrosinase in aqueous medium increased the scavenging activity of oxidation products [7]. In a previous study, the IC_50_ value of ascorbic acid (Vitamin C) was found to be almost 31 µM [34,35]. In comparison with ascorbic acid, it was found that FA products showed higher antioxidant capacities than ascorbic acid, confirming their potential as promising antioxidant agents. Another study reported the use of fungal laccase in edible vegetable oils to save from the negative effect of oxidation by enzymatic oxidation products of phenols [5].

### 3.7. Anti-Proliferative Activity of FA Products

The anti-proliferative activity of the FA molecule and its oxidation products was determined at different concentrations over 48 h against Caco-2 cells using neutral red uptake (NRU) method. Results were presented as IC_50_ values with standard deviations. It was found that 1% (*v*/*v*) DMSO did not show any toxic effect against Caco2 cells.

As presented in Table 4, presenting IC_50_ values for FA and its oxidation products (*p* ˃ 0.05), FA products P2, P3, P6 and P7 presented higher toxic effect against colon cells than FA, while FA products P1 and P5 had a less toxic effect against colon cells compared to FA. Furthermore, FA and its product P4 presented a similar effect against colon cells. These findings can be due to antiradical activities of FA-oxidation products. Indeed, the antioxidant effect of phenols is the main raison of their cytotoxic effect on tumor cells. Normal cells are usually sensitive to ROS, and FA could prevent neuronal cell systems from ROS-mediated damage [36]. It was noted that tumor cells may be affected by H_2_O_2_ and H_2_O_2_ deprivation has caused cell cycle arrest or apoptosis [37]. For example, it was found that the FA molecule at 1.5 mM decreased the number of Caco2 cells to 43% after 48 h of treatment [38].

Finally, the results confirmed that the enzymatic oligomerization of FA could produce two dehydromers and two tetramers (P2, P3, P6 and P7) with important anti-proliferative activity against tumor cells due to their important antiradical activities compared to FA.

## 4. Conclusions

In the present work, FA-laccase-catalyzed oxidation in aqueous medium led to several products of which seven products were identified. The oxidation products that were investigated in this study included four dehydrodimers (P1, P2, P3, P5) at MM = 386 g/mol, two dehydrotetramers (P6, P7) at MM = 770 g/mol and one decarboxylated dehydrodimer (P4) at MM = 340 g/mol. Structural characterization results showed that FA-dehydrodimers (P1, P5) were symmetric dimers of two FA-monomers covalently bound through a C_8_-C_8_ linkage. Additionally, FA-dehydrodimers (P2, P3, P4) were asymmetric dimers of two FA-monomers covalently bound through a C_8_-C_5_ linkage (P2, P4) or a C_8_-O-C_4_ linkage (P3). Physicochemical characterization findings showed that the oxidation products presented a higher lipophilicity than that of FA. Moreover, symmetric dimers and tetra dimers had a higher melting point compared to FA and its asymmetric dimers, indicating a higher force of attraction between atoms for symmetric dimers and tetra dimers. Antioxidant and anti-proliferative properties indicated that enzymatic oligomerization increased antioxidant and anti-proliferative properties for P2, P3 and P6 compared to FA due to an increase in electron donating groups after the dimerization process, and the carboxylic group with an unsaturated C-C double bond. These products with good activities (antioxidant, anti-proliferative) could be used in in biotechnological domains such as antioxidant additives for food preservation.

## Figures and Tables

**Table 1:** ^1^H and ^13^C NMR data of FA-dehydrodimers of symmetric P1, P5 at 300 MHz in Dimethyl sulfoxide (DMSO).

A	P1		P5	
Position	δ^1^H (ppm)	δ^13^C	δ^1^H (ppm)	δ^13^C
A1, B1	-	130.1	-	130.6
A2, B2	6.79 (2H)	107.6	7.06 (2H)	109.5
A3, B3	-	146.5	-	146.8
A4, B4	-	147.2	-	147.8
A5, B5	6.93 (2H)	115.1	6.96 (2H)	116.3
A6, B6	6.84 (2H)	117.5	6.83 (2H)	118.2
A7, B7	5.69 (2H)	82.5	5.78 (2H)	82.9
A8, B8	3.56 (2H)	48.6	4.08 (2H)	49.1
OMe A3, B3	3.91 (3H)	56.4	3.87 (2H)	56.7
OH A4, B4	5.86 (2H)	-	5.91 (2H)	-
A9, B9	-	174.5	9.04	173.1

**Table 2:** ^1^H and ^13^C NMR data of FA-dehydrodimers of asymmetric P2, P3, P4 (B) at 300 MHz in Dimethyl sulfoxide (DMSO).

B	P2		P3		P4	
Position	δ^1^H (ppm)	δ^13^C	δ^1^H (ppm)	δ^13^C	δ^1^H (ppm)	δ^13^C
A1	-	129.3	-	129.8	-	128.5
A2	6.99	112.42	6.85	110.8	7.39 (1H)	110.9
A5	-	128.07	6.9	127.1	-	125.8
A6	7.02 (1H)	113.17	7.15	115.2	7.32 (1H)	115.9
A7	7.68	146.03	-	149.4	6.88	150.0
A8	6.3	117.2	6.5	119.1	7.33 (1H)	118.6
B1	-	131.67	-	129.3	-	131.4
B2	7.17 (1H)	108.41	7.1	106.7	7.32 (1H)	105.6
B5	6.99 (1H)	116.7	6.89	115.8	6.92 (1H)	116.2
B6	7.23 (1H)	117.7	7.3	118.1	7.44 (1H)	121.3
B7	5.84 (1H)	86.2	5.79	86.9	-	88.1
B8	3.86 (1H)	53.31	-	52.6	3.75	56.4
A3	-	145.3	-	147.2	-	146.5
B3	-	148.8	-	147.8	-	148.0
A4	-	146.2	-	147.6	-	147.9
B4	-	146.6	-	149.1	-	148.5
OMe A3	3.86 (3H)	55.9	3.82	54.9	3.87 (3H)	55.84
OMe B3	3.86 (3H)	55.95	3.80	55.6	3.82 (3H)	55.93
OH A4	-	-	-	-	9.86 (1H)	-
OH B4	9.01	-	8.99	-	9.69	-
A9	9.01	168.3	-	166.9	9.05	169.4
B9	8.98	172.9	-	169.3	-	-

**Table 3:** Log*P* and melting point values (°C) of FA-Laccase-catalyzed oxidation products.

	Log*P*	Melting Point (°C)
FA	1.45 ± 0.04 ^f^	173.4 ± 3.3 ^c^
P1	1.51± 0.02 ^e^	183.5 ± 4.1 ^b^
P2	1.59 ± 0.02 ^d^	155.8 ± 4.3 ^d^
P3	1.67 ± 0.05 ^c^	136.4 ± 3.7 ^e^
P4	1.74 ± 0.04 ^b^	149.4 ± 2.6 ^d^
P5	1.79 ± 0.06 ^b^	201.2 ± 3.4 ^a^
P6	1.93 ± 0.06 ^a^	206.9 ± 2.9 ^a^
P7	1.96 ± 0.04 ^a^	203.5 ± 1.6 ^a^

Each value is presented as mean ± standard deviation (n = 3). Values in each column with different letter are significantly different at the 0.05% level (Duncan’s test).

**Table 4:** Antiradical (IC_50_, TEAC coefficient) and anti-proliferative (IC_50_) activities of FA and its oxidation products.

	Antiradical Activity (ABTS)		Anti-Proliferative Activity (Neutral Red) Assay
	IC_50_ (µM)	TEAC	IC_50_ (mM)
FA	7.2 ± 0.1 ^e^	1.24 ± 0.04 ^b^	3.6 ± 0.3 ^c^
P1	24.4 ± 0.8 ^a^	0.36 ± 0.04 ^d^	7.9 ± 0.4 ^a^
P2	6.4 ± 0.2 ^f^	1.39 ± 0.05 ^a^	1.8 ± 0.2 ^f^
P3	6.6 ± 0.3 ^f^	1.35 ± 0.04 ^a^	2.1 ± 0.2 ^e^
P4	19.9 ± 1.1 ^b^	0.45 ± 0.05 ^d^	3.2 ± 0.3 ^c^
P5	11.9 ± 0.4 ^c^	0.75 ± 0.05 ^c^	4.7 ± 0.3 ^b^
P6	6.5 ± 0.2 ^f^	1.37 ± 0.03 ^a^	2.1 ± 0.1 ^e^
P7	7.1 ± 0.1 ^e^	1.26 ± 0.04 ^b^	2.5 ± 0.2 ^d^
Trolox	8.9 ± 0.2 ^d^	1	-

Each value is presented as mean ± standard deviation (n = 3). Values which do not have the same letter in each column are significantly different at the 0.05% level (Duncan’s test).

## Data Availability

Not applicable.
